# Modulation of Nuclear Factor Kappa B Signaling and microRNA Profiles by Adalimumab in LPS-Stimulated Keratinocytes

**DOI:** 10.3390/ijms262010035

**Published:** 2025-10-15

**Authors:** Aleksandra Plata-Babula, Wojciech Kulej, Paweł Ordon, Julia Gajdeczka, Martyna Stefaniak, Artur Chwalba, Piotr Gościniewicz, Tomasz Kulpok, Beniamin Oskar Grabarek

**Affiliations:** 1 Collegium Medicum, WSB University, 41-300 Dąbrowa Górnicza, Poland; 2 Faculty of Medicine and Health Sciences, Andrzej Frycz Modrzewski University in Cracow, 30-705 Cracow, Poland; 3 General Rehabilitation Sub-Department, Edmund Wojtyla Małopolski Hospital for Lung Diseases and Rehabilitation, 32-310 Jaroszowiec, Poland; 4 Department of Pharmacology, Faculty of Medical Sciences in Zabrze, Medical University of Silesia in Katowice, 41-808 Zabrze, Poland

**Keywords:** adalimumab, NF-κB, psoriasis, lipopolysaccharide, microRNA, STRING, immunomodulation

## Abstract

Psoriasis is a chronic inflammatory skin disease characterized by keratinocyte hyperactivation and dysregulated cytokine signaling, with nuclear factor kappa B (NF-κB), a master transcription factor that regulates immune and inflammatory gene expression, playing a central role. Adalimumab, a monoclonal antibody that inhibits tumor necrosis factor alpha (TNF-α), is widely used in psoriasis therapy, yet its molecular effects on NF-κB-associated genes and microRNAs (miRNAs) in keratinocytes remain insufficiently defined. In this study, immortalized human keratinocytes (HaCaT cells) were exposed to lipopolysaccharide (LPS) to induce inflammatory stress and treated with adalimumab for 2, 8, and 24 h. Transcriptome-wide profiling was performed using messenger RNA (mRNA) and miRNA microarrays, followed by validation with reverse transcription quantitative polymerase chain reaction (RT-qPCR) and enzyme-linked immunosorbent assay (ELISA). Bioinformatic analyses included prediction of miRNA–mRNA interactions, construction of protein–protein interaction (PPI) networks, and gene ontology (GO) enrichment. Adalimumab reversed LPS-induced upregulation of NF-κB-associated genes, including inhibitor of nuclear factor kappa-B kinase subunit beta (IKBKB), interleukin-1 receptor-associated kinase 1 (IRAK1), TNF receptor-associated factor 2 (TRAF2), mitogen-activated protein kinase kinase kinase 7 (MAP3K7), and TNF alpha-induced protein 3 (TNFAIP3), with concordant changes observed at the protein level. Several regulatory miRNAs, notably miR-1297, miR-30a, miR-95-5p, miR-125b, and miR-4329, showed reciprocal expression changes consistent with anti-inflammatory activity. STRING analysis identified IKBKB as a central hub in the PPI network, while GO enrichment highlighted immune regulation, apoptosis, and NF-κB signaling. These findings demonstrate that adalimumab modulates NF-κB activity in keratinocytes through coordinated regulation of gene, protein, and miRNA expression, providing mechanistic insight into TNF-α blockade in psoriasis.

## 1. Introduction

Psoriasis is a common, chronic, immune-mediated inflammatory skin disorder that affects approximately 2–3% of the global population and imposes a substantial physical, psychological, and socioeconomic burden [1]. It is clinically characterized by well-demarcated, erythematous plaques with silvery scales, most frequently affecting the scalp, elbows, knees, and lower back [2,3]. Although psoriasis primarily manifests in the skin, it is now recognized as a systemic disease associated with a broad spectrum of comorbidities, including psoriatic arthritis, metabolic syndrome, cardiovascular disorders, and mental health complications such as depression and suicidality [4].

Over the past two decades, therapeutic strategies for psoriasis have evolved from nonspecific immunosuppression to targeted molecular therapies [5,6]. Traditional approaches—including topical corticosteroids, vitamin D analogs, phototherapy, and systemic agents such as methotrexate or cyclosporine—provide varying degrees of symptom control. The advent of biologic therapies, monoclonal antibodies specifically designed to neutralize proinflammatory cytokines, has revolutionized disease management [3,7]. Among these, tumor necrosis factor alpha (TNF-α) inhibitors (adalimumab, etanercept, infliximab) and newer agents targeting interleukin (IL) (IL-12/23) (ustekinumab), IL-17 (secukinumab, ixekizumab), and IL-23 (guselkumab, risankizumab) have demonstrated remarkable clinical efficacy and safety in moderate-to-severe plaque psoriasis [8]. Despite these advances, long-term disease control remains challenging due to primary non-responsiveness, secondary resistance, and relapse after treatment discontinuation [1,9,10].

Adalimumab, a monoclonal antibody against TNF-α, is highly effective in moderate-to-severe plaque psoriasis and psoriatic arthritis, improving Psoriasis Area and Severity Index (PASI) scores and patient quality of life [11]. By neutralizing TNF-α, it downregulates key inflammatory cascades, including nuclear factor kappa B (NF-κB), mitogen-activated protein kinases (MAPK), and Janus kinase/signal transducer and activator of transcription (JAK/STAT) signaling. However, the precise molecular effects of adalimumab on keratinocyte signaling under inflammatory stress remain incompletely defined [12,13,14].

At the molecular level, NF-κB acts as a central hub integrating upstream cytokine signals with downstream transcriptional responses in psoriasis [15,16]. NF-κB comprises transcription factors RelA (p65), RelB, c-Rel, p50, and p52, which are normally sequestered in the cytoplasm by inhibitors of κB (IκBs). Upon stimulation by TNF-α, IL-1β, or microbial products such as lipopolysaccharide (LPS), the IκB kinase (IKK) complex phosphorylates IκBα, leading to its proteasomal degradation. This releases NF-κB dimers to translocate into the nucleus, where they activate genes involved in inflammation, immune cell recruitment, angiogenesis, and cell survival [17,18].

In psoriatic skin, NF-κB signaling is constitutively activated in both keratinocytes and infiltrating immune cells, driving sustained expression of cytokines (e.g., IL-6, IL-8), chemokines (e.g., C-C motif chemokine ligand 20; CCL20), and antimicrobial peptides (e.g., psoriasin (S100A7) and human β-defensin-2 (hBD-2)) [19,20,21,22]. Moreover, NF-κB synergizes with Signal Transducer and Activator of Transcription 3 (STAT3), Activator Protein 1 (AP-1), and MAPK pathways, amplifying the inflammatory loop characteristic of chronic disease [19,20].

NF-κB also functions as both a driver and effector within the TNF-α/IL-23/IL-17 axis, the dominant pathogenic pathway in plaque psoriasis [23,24]. TNF-α activates NF-κB in keratinocytes and promotes IL-23 production in dendritic cells, sustaining Th17 differentiation and IL-17 secretion. IL-17A and TNF-α co-activate NF-κB, further enhancing keratinocyte inflammatory responses and fueling disease progression [25,26,27,28]. The genetic landscape of psoriasis reinforces the importance of NF-κB. Susceptibility genes such as NFKBIA (IκBα), TNFAIP3 (A20), TNIP1, and CARD14 regulate NF-κB activation or inhibition [29]. Gain-of-function mutations in Caspase Recruitment Domain Family Member 14 (CARD14) result in constitutive NF-κB activity in keratinocytes and psoriasis-like inflammation in murine models [30,31]. Parallel to this, microRNAs (miRNAs)—short, non-coding RNAs that regulate gene expression post-transcriptionally—are increasingly recognized as key regulators of cutaneous homeostasis and immune balance in psoriasis [32]. Aberrant miRNA profiles have been reported in psoriatic lesional skin, serum, and immune cell subsets [33,34,35,36]. Several miRNAs modulate NF-κB activity by targeting upstream or downstream effectors, including IKKβ, IκBα, and p65 [37,38,39]. Integration of miRNA biology into psoriasis pathogenesis provides novel opportunities for biomarker discovery and therapeutic targeting [40,41,42,43]. However, the effect of TNF-α inhibition on the keratinocyte gene–miRNA axis remains poorly understood.

Keratinocytes, once considered passive bystanders, are now recognized as active participants in psoriatic inflammation. In response to TNF-α, IL-17A, and pathogen-associated molecular patterns (PAMPs) such as lipopolysaccharide (LPS), a Toll-like receptor 4 (TLR4) agonist, they produce pro-inflammatory cytokines, chemokines, and antimicrobial peptides, thereby amplifying local immune responses [21,44,45,46].

In this study, LPS was used to induce an inflammatory environment in keratinocytes in order to mimic the cellular stress conditions characteristic of psoriatic skin [46,47]. LPS is a well-defined TLR4 agonist that activates innate immune pathways [48], leading to the rapid induction of pro-inflammatory cytokines and chemokines such as TNF-α, IL-1β, IL-6, and C-X-C motif chemokine ligand (CXCL8), as well as activation of the NF-κB and MAPK signaling cascades [21,44,45,46]. These molecular events closely parallel those observed in psoriatic epidermis, where keratinocytes exhibit enhanced responsiveness to inflammatory stimuli and sustained NF-κB activation [21]. Clinically, elevated expression of TLR4 has been detected in psoriatic lesions and in the peripheral blood of affected patients, suggesting that microbial components and endotoxins may amplify local inflammation and contribute to disease exacerbation [49,50]. Therefore, the use of LPS in vitro provides a reproducible and controllable model for studying keratinocyte-driven inflammation [51] and allows for precise evaluation of how adalimumab [52,53,54] modulates NF-κB-dependent transcriptional and post-transcriptional responses under conditions of innate immune activation [47]. This approach facilitates the dissection of molecular mechanisms underlying TNF-α blockade in epithelial cells, which are key contributors to the inflammatory milieu in psoriasis [21].

Although the NF-κB signaling pathway has been extensively characterized in keratinocytes, most studies have focused on canonical activation mechanisms or short-term inflammatory responses. However, the temporal dynamics and gene–miRNA interactions underlying NF-κB modulation during prolonged inflammatory stimulation remain poorly understood. Our study provides a comprehensive temporal analysis of NF-κB-related transcriptional and post-transcriptional responses in HaCaT cells exposed to LPS and adalimumab, thereby expanding the molecular landscape of NF-κB regulation in epidermal inflammation.

Therefore, in this study we aimed to investigate how adalimumab modulates the expression of NF-κB pathway-associated genes and their regulatory miRNAs in an LPS-stimulated human immortalized keratinocyte (HaCaT) model.

## 2. Results

### 2.1. Cytotoxicity Results

At the concentrations selected for the expression analyses—LPS 1 μg/mL and adalimumab 8 μg/mL—cell viability remained above 90%, indicating no significant cytotoxicity (*p* > 0.05, one-way analysis of variance ANOVA). Only the supratherapeutic adalimumab concentration (80 μg/mL) reduced viability to ~41% of control (*p* < 0.05, Tukey post hoc).

Based on these results, keratinocytes were stimulated with LPS (1 μg/mL) for 8 h to model an inflammatory state [55], thereafter, adalimumab (8 μg/mL; Humira^®^, AbbVie, North Chicago, IL, USA)—a concentration corresponding to therapeutic plasma levels in psoriasis patients [55,56]—was added for 2, 8, or 24 h. Untreated cells served as negative controls, whereas cells exposed only to LPS were used as inflammatory controls [55,56].

### 2.2. Transcriptomic Profiling Reveals LPS-Induced NF-κB Activation and Its Attenuation by Adalimumab

Genome-wide microarray analysis revealed extensive transcriptional reprogramming of NF-κB-related genes in HaCaT keratinocytes following LPS stimulation and subsequent adalimumab treatment. Differentially expressed genes (DEGs) were defined by a fold change (|FC|) ≥ 2.0 and adjusted *p* < 0.05.

As expected, LPS stimulation strongly activated the NF-κB pathway compared with untreated controls (C), resulting in the upregulation of canonical signaling mediators such as *IKBKB*, *IRAK1*, *TRAF2*, *SYK*, *MAP3K7*, *TNF*, and *CXCL2*. In addition, several genes associated with anti-apoptotic activity and feedback inhibition of inflammation—including *BIRC2*, *BIRC3*, *XIAP*, and *TNFAIP3*—were significantly elevated, reflecting a robust pro-inflammatory and survival response.

Adalimumab markedly counteracted these LPS-induced changes at all examined time points (2 h, 8 h, and 24 h post-treatment), indicating consistent inhibition of NF-κB–dependent transcription. Specifically, 14 DEGs were identified in the H_2_ vs. LPS comparison, 14 DEGs in H_8_ vs. LPS, and 10 DEGs in H_24_ vs. LPS (*p* < 0.05). Early suppression (H_2_) was most pronounced for *TNFAIP3*, *BCL2L1*, *TRAF2*, and *MAP3K7*, whereas prolonged exposure (H_8_ and H_24_) led to broader inhibition encompassing *CXCL2*, *BIRC2*, and *BIRC3*. Downregulation of signal transducers such as *SYK*, *TAB1*, and *TAB2* further confirmed that adalimumab attenuates NF-κB activation at multiple regulatory levels and time points.

**Figure 1:**
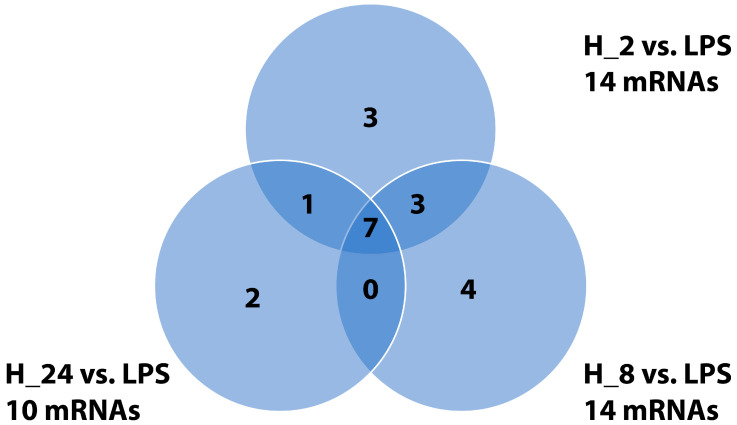
Venn diagram showing overlap and unique sets of differentially expressed NF-κB–related genes in HaCaT keratinocytes treated with adalimumab for 2 h (H_2_), 8 h (H_8_), and 24 h (H_24_) after LPS stimulation. Each circle represents DEGs identified at a given time point (|fold change| ≥ 2.0; adjusted *p* < 0.05). Overlapping regions indicate shared genes among time points. Data derived from three independent biological replicates (*n* = 3). Abbreviations: LPS, lipopolysaccharide-stimulated cells; H_2_, H_8_, H_24_, adalimumab treatment for 2, 8, and 24 h, respectively.

**Figure 2:**
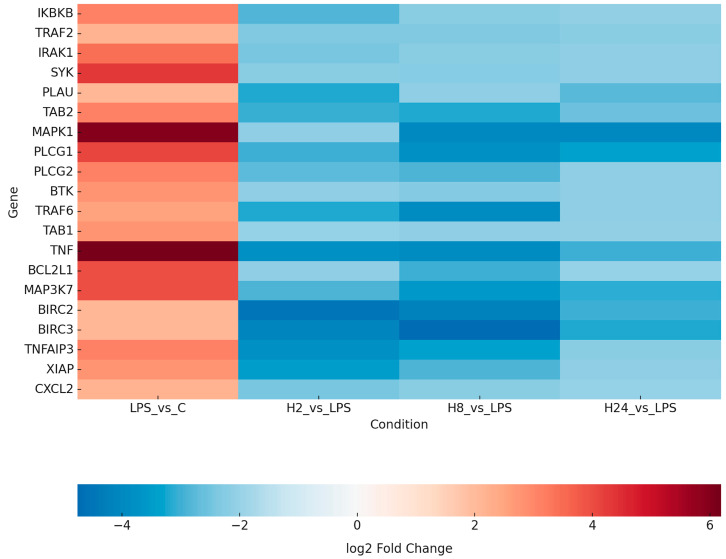
Heatmap of NF-κB–related gene expression in HaCaT keratinocytes determined by microarray analysis. Data represent relative expression values for untreated control (C), LPS-stimulated cells, and adalimumab-treated cells (H_2_, H_8_, H_24_). Comparisons: LPS vs. C (inflammatory induction) and H_2_/H_8_/H_24_ vs. LPS (adalimumab effects). Data are presented as mean values from three biological replicates (*n* = 3). Abbreviations: C, untreated control; LPS, lipopolysaccharide-stimulated cells; H_2_, H_8_, H_24_, adalimumab treatment for 2, 8, and 24 h, respectively. Gene abbreviations: *IKBKB*, Inhibitor of Nuclear Factor Kappa B Kinase Subunit Beta; *TRAF2*, TNF Receptor Associated Factor 2; *IRAK1*, Interleukin-1 Receptor-Associated Kinase 1; *SYK*, Spleen Tyrosine Kinase; *PLAU*, Plasminogen Activator, Urokinase; *TAB2*, TGF-Beta Activated Kinase 1 Binding Protein 2; *MAPK14*, Mitogen-Activated Protein Kinase 14 (p38α); *PLCG1*, Phospholipase C Gamma 1; *PLCG2*, Phospholipase C Gamma 2; *BTK*, Bruton’s Tyrosine Kinase; *TRAF6*, TNF Receptor Associated Factor 6; *TAB1*, TGF-Beta Activated Kinase 1 Binding Protein 1; *TNF*, Tumor Necrosis Factor; BCL2L1, BCL2 Like 1 (Bcl-xL); *CXCL2*, C-X-C Motif Chemokine Ligand 2; *MAP3K7*, Mitogen-Activated Protein Kinase Kinase Kinase 7 (TAK1); *BIRC2*, Baculoviral IAP Repeat Containing 2 (cIAP1); *BIRC3*, Baculoviral IAP Repeat Containing 3 (cIAP2); *TNFAIP3*, Tumor Necrosis Factor Alpha-Induced Protein 3 (A20); *XIAP*, X-Linked Inhibitor of Apoptosis Protein.

The Venn diagram (Figure 1) illustrates the overlap and time-specific distribution of NF-κB-related DEGs following adalimumab treatment. The strongest intersection among all treatment times was observed for *TRAF6*, *TAB1*, and *TNF*, suggesting persistent modulation of these central NF-κB components. The heatmap (Figure 2) provides a global visualization of transcriptional changes across all experimental conditions. The complete list of DEGs for each time point is provided in Supplementary Table S1, while the subset of key transcripts validated by RT-qPCR and ELISA is summarized in Table 1.

A complete list of all differentially expressed genes identified by microarray analysis is provided in Supplementary Table S1. In the main manuscript, Table 1 presents only those genes that were further validated at the mRNA level by RT-qPCR and whose protein products were quantified using ELISA, reflecting the core molecular targets analyzed in this study.

Gene abbreviations: *CXCL2*, C-X-C Motif Chemokine Ligand 2; *MAP3K7*, Mitogen-Activated Protein Kinase Kinase Kinase 7; *BIRC2*, Baculoviral IAP Repeat Containing 2; *BIRC3*, Baculoviral IAP Repeat Containing 3; *TNFAIP3*, Tumor Necrosis Factor Alpha-Induced Protein 3; *XIAP*, X-Linked Inhibitor of Apoptosis Protein.

### 2.3. Validation of NF-κB mRNA Expression Using RT-qPCR

To validate the microarray data, RT-qPCR was performed for seven representative NF-κB-related genes: *TNFAIP3*, *BCL2L1*, *CXCL2*, *MAP3K7*, *BIRC2*, *BIRC3*, and *XIAP*.

Consistent with the transcriptomic findings, LPS stimulation significantly increased the expression of all analyzed genes compared with untreated controls, confirming activation of the NF-κB pathway. Subsequent adalimumab treatment led to a pronounced and time-dependent downregulation of these transcripts relative to LPS-treated cells.

**Figure 3:**
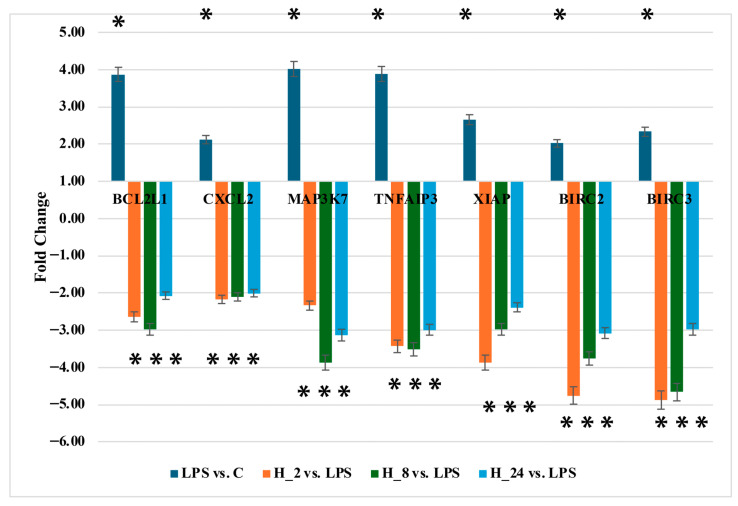
RT-qPCR validation of selected NF-κB-related genes in HaCaT keratinocytes. Bar plots show relative mRNA expression (fold change) of TNFAIP3, BCL2L1, CXCL2, MAP3K7, BIRC2, BIRC3, and XIAP in untreated control (C), LPS-stimulated cells, and adalimumab-treated cells (H_2_, H_8_, H_24_). LPS stimulation significantly increased transcript levels compared with control, whereas adalimumab treatment reduced expression relative to LPS. Data represent mean ± SEM from three independent biological replicates (*n* = 3). Statistical analysis: one-way ANOVA followed by Scheffe’s post hoc test. Asterisks indicate differential regulation relative to the reference condition: *p* < 0.05 vs. control (C) for LPS, and *p* < 0.05 vs. LPS for H_2_, H_8_, and H_24_. Abbreviations: C, untreated control; LPS, lipopolysaccharide-stimulated cells; H_2_, H_8_, H_24_, adalimumab treatment for 2, 8, and 24 h, respectively. Gene abbreviations: *BCL2L1*, BCL2 Like 1; *CXCL2*, C-X-C Motif Chemokine Ligand 2; *MAP3K7*, Mitogen-Activated Protein Kinase Kinase Kinase 7; *BIRC2*, Baculoviral IAP Repeat Containing 2; *BIRC3*, Baculoviral IAP Repeat Containing 3; *TNFAIP3*, Tumor Necrosis Factor Alpha-Induced Protein 3; *XIAP*, X-Linked Inhibitor of Apoptosis Protein.

The strongest inhibitory effects were observed for *TNFAIP3* and *BCL2L1* at 2 h and 8 h, whereas *CXCL2* and *MAP3K7* exhibited gradual reductions across all time points. *BIRC2*, *BIRC3*, and *XIAP* followed similar patterns of suppression, closely mirroring the microarray results. Although fold-change magnitudes differed between platforms, the direction of expression changes was consistent (Figure 3). These results confirm that adalimumab effectively reverses LPS-induced NF-κB activation at the transcriptional level.

### 2.4. Profiling of miRNA Expression Levels

Microarray profiling identified several microRNAs predicted to target NF-κB-associated transcripts affected by adalimumab. Differential expression was defined by |fold change| ≥ 2 and *p* < 0.05 across all experimental groups.

LPS stimulation downregulated miRNAs predicted to inhibit inflammatory mediators, including miR-1297 and miR-30a (targeting MAP3K7), miR-95-5p (targeting CXCL2), and miR-125b (targeting TNFAIP3). Conversely, miR-4329 (targeting XIAP) and miR-20b-3p (targeting BIRC3) were upregulated, indicating enhanced pro-survival signaling.

Adalimumab treatment reversed these trends in a time-dependent manner. Anti-inflammatory miRNAs (miR-1297, miR-30a, miR-95-5p, miR-125b) were significantly restored, whereas pro-survival miRNAs (miR-4329, miR-20b-3p) were downregulated compared with LPS. These reciprocal expression patterns suggest regulatory interactions consistent with the mRNA profiles and imply that adalimumab exerts post-transcriptional control over NF-κB signaling through miRNA modulation (Table 2).

### 2.5. Quantification of BCL2L1, CXCL2, MAP3K7, BIRC2, BIRC3, TNFAIP3, and XIAP Protein Levels

ELISA quantification confirmed that LPS stimulation significantly increased protein levels of NF-κB and MAPK pathway components, including BCL2L1/Bcl-xL, CXCL2, MAP3K7, BIRC2, BIRC3, TNFAIP3/A20, and XIAP.

Adalimumab administration led to a marked reduction in the concentration of all these proteins, consistent with transcript-level findings. For instance, BCL2L1 levels declined from 87.12 ± 19 ng/mL (LPS) to approximately 17–18 ng/mL after treatment, returning close to baseline. CXCL2 and MAP3K7 were also significantly reduced, while BIRC2 and BIRC3 decreased more than twofold compared with LPS-treated cells. TNFAIP3 and XIAP followed a similar trend, though XIAP remained slightly above control levels (Table 3).

Together, these data confirm that adalimumab mitigates NF-κB activation in keratinocytes by suppressing both transcriptional and translational outputs of key pathway mediators.

### 2.6. Protein–Protein Interaction (PPI) Network Analysis via STRING Database

**Figure 4:**
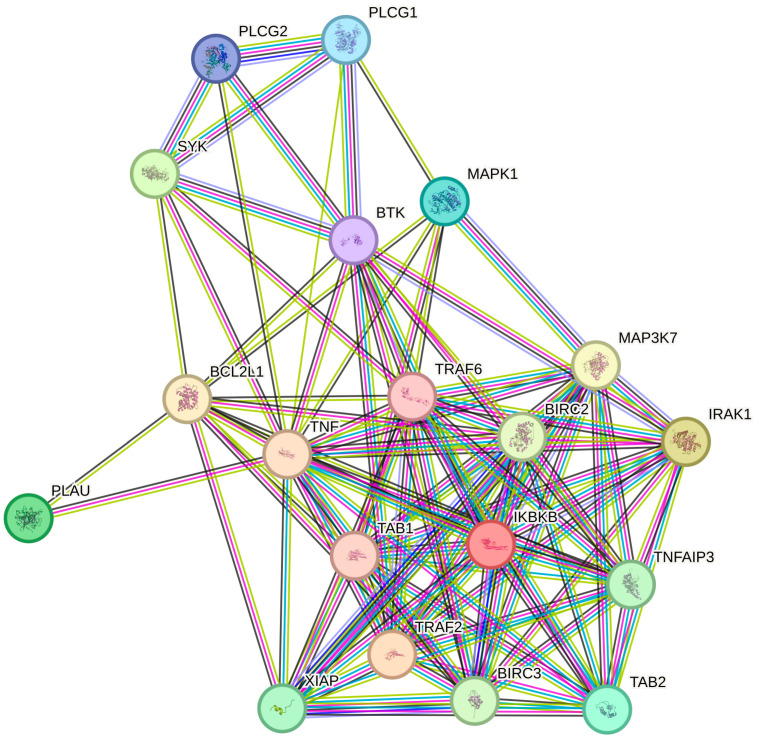
STRING-based PPI network for NF-κB–related genes from Table 2. Protein abbreviations: IKBKB, Inhibitor of Nuclear Factor Kappa B Kinase Subunit Beta; TRAF2, TNF Receptor Associated Factor 2; IRAK1, Interleukin-1 Receptor-Associated Kinase 1; SYK, Spleen Tyrosine Kinase; PLAU, Plasminogen Activator, Urokinase; TAB2, TGF-Beta Activated Kinase 1 (MAP3K7) Binding Protein 2; MAPK14, Mitogen-Activated Protein Kinase 14; PLCG1, Phospholipase C Gamma 1; PLCG2, Phospholipase C Gamma 2; BTK, Bruton’s Tyrosine Kinase; TRAF6, TNF Receptor Associated Factor 6; TAB1, TGF-Beta Activated Kinase 1 (MAP3K7) Binding Protein 1; TNF, Tumor Necrosis Factor; BCL2L1, BCL2 Like 1; CXCL2, C-X-C Motif Chemokine Ligand 2; MAP3K7, Mitogen-Activated Protein Kinase Kinase Kinase 7; BIRC2, Baculoviral IAP Repeat Containing 2; BIRC3, Baculoviral IAP Repeat Containing 3; TNFAIP3, Tumor Necrosis Factor Alpha-Induced Protein 3; XIAP, X-Linked Inhibitor of Apoptosis Protein.

STRING-based PPI analysis demonstrated that NF-κB-related DEGs formed a densely interconnected network of 20 nodes and 101 edges—far exceeding the 16 edges expected by chance (*p* < 1 × 10^−16^). IKBKB (IKKβ) emerged as the central hub connecting multiple upstream regulators (TRAF2, TRAF6, IRAK1, MAP3K7, TNF), underscoring its pivotal role in coordinating inflammatory signaling within keratinocytes (Figure 4).

**Figure 5:**
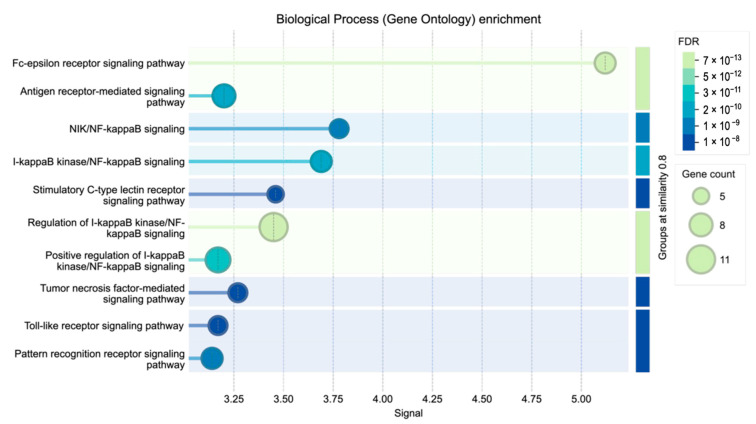
Gene Ontology (GO:BP) enrichment for downregulated DEGs from Table 2. Differentially expressed genes were identified in LPS vs. untreated control (C), and their downregulation following adalimumab treatment (H_2_, H_8_, H_24_ vs. LPS) was analyzed using the STRING enrichment tool (FDR < 0.05). Terms were grouped by semantic similarity (threshold = 0.8) and ranked by enrichment strength. Only the top 10 processes are shown; the complete list is provided in Table A1.

Gene Ontology (GO:BP) enrichment of downregulated genes revealed significant associations with cytokine-mediated signaling, regulation of apoptosis, and innate immune responses (FDR < 0.05). The top 10 enriched biological processes are shown in Figure 5, and the full list is provided in Appendix A (Table A1). These findings indicate that adalimumab exerts broad anti-inflammatory effects by targeting core NF-κB nodes and functionally linked processes.

To further characterize the biological relevance of the transcriptional changes, downregulated DEGs were subjected to Gene Ontology (GO:BP) enrichment analysis in STRING. The top enriched processes, summarized in Figure 5, included cytokine-mediated signaling, regulation of apoptosis, and innate immune responses (FDR < 0.05). A complete list of enriched processes with corresponding genes is provided in Table A1.

## 3. Discussion

The present study contributes novel insights into the NF-κB signaling network in HaCaT cells by integrating time-dependent transcriptomic, miRNA, and protein-level analyses. Unlike previous reports focusing on single endpoints or isolated cytokines, we identified coordinated changes in both NF-κB target genes and regulatory microRNAs following adalimumab exposure, revealing potential feedback mechanisms that modulate inflammation resolution. This integrative approach expands the NF-κB knowledge base for keratinocytes and provides a reference for future therapeutic studies targeting chronic cutaneous inflammation. Previous reports have established the central role of TNF-α in activating NF-κB signaling and sustaining psoriatic inflammation [11,23,24]; our findings extend this knowledge by revealing how TNF-α blockade reprograms keratinocyte transcriptional and post-transcriptional networks.

Consistent with earlier studies showing that TNF-α is a potent activator of NF-κB [21,57,58], we found that LPS stimulation in HaCaT keratinocytes led to significant upregulation of *IKBKB*, *IRAK1*, *TRAF2*, *CXCL2*, *MAP3K7*, *BCL2L1*, *BIRC2*, *BIRC3*, *XIAP*, and *TNFAIP3*. These genes are known to drive cytokine production, immune cell recruitment, and resistance to apoptosis—key elements in the pathogenesis of psoriatic inflammation [21,57,58]. Importantly, our data showed that adalimumab consistently downregulated these transcripts across all time points, supporting its broad anti-inflammatory action through inhibition of TNF-α-mediated signaling [59].

A closer examination of individual gene responses in our dataset further illustrates this effect. For example, we observed that *BCL2L1* was markedly upregulated after LPS stimulation and subsequently reduced by adalimumab. Since *BCL2L1* promotes cell survival by inhibiting apoptosis [60], this suggests that adalimumab may restore apoptotic balance and contribute to normalization of epidermal architecture, in agreement with previous reports [61,62].

Similarly, our experiments revealed that *CXCL2* was strongly upregulated by LPS and suppressed by adalimumab at both mRNA and protein levels. This indicates inhibition of acute inflammatory signaling and attenuation of neutrophil recruitment—pathways previously linked to psoriatic lesion activity [63,64,65,66,67].

We also found that *MAP3K7 (TAK1)*, a central kinase upstream of NF-κB and MAPK pathways [68], was significantly upregulated by LPS but downregulated by adalimumab. This highlights MAP3K7 inhibition as a potential mechanism by which TNF-α blockade disrupts inflammatory cascades, consistent with earlier mechanistic insights [69,70,71].

In addition, our results showed that apoptosis regulators *BIRC2* and *BIRC3* were induced by LPS and suppressed by adalimumab, supporting the concept that TNF-α inhibition corrects aberrant survival signaling in keratinocytes [72,73]. Likewise, *TNFAIP3 (A20)* was upregulated under inflammatory stress in our model, likely reflecting a compensatory feedback mechanism [74,75]. Similarly, TNFAIP3 (A20) is a well-established negative-feedback regulator of NF-κB signaling [76,77]. In our model, LPS robustly increased TNFAIP3, consistent with compensatory feedback to excessive IKK/NF-κB activity, whereas adalimumab reduced TNFAIP3 expression across time points. This pattern suggests diminished upstream inflammatory drive under TNF-α blockade, in line with prior observations [78,79]. Thus, lower A20 after adalimumab reflects reduced pathway stimulation rather than a pro-inflammatory effect.

Interestingly, we found that *XIAP* remained moderately elevated after adalimumab treatment despite a partial reduction from LPS-induced levels. This persistence may reflect residual protective signaling in keratinocytes, and further studies are warranted to clarify its role in treatment response [80,81,82].

Taken together, our data support the concept that adalimumab exerts dual effects in keratinocytes—downregulating proinflammatory cytokine networks and aberrant survival pathways—thereby promoting resolution of inflammation and restoration of epidermal homeostasis [52,83,84,85,86].

In parallel with these transcriptomic and proteomic findings, we demonstrated that adalimumab modulates miRNA expression. These changes likely contribute to fine-tuning of inflammatory and apoptotic gene networks. Previous studies have linked miRNAs to NF-κB regulation in keratinocytes [57,87,88,89], but our study adds novel evidence for specific miRNA–mRNA pairs influenced by adalimumab.

Specifically, we observed that miR-1297 and miR-30a, both suppressed by LPS, were restored by adalimumab. These miRNAs are predicted to target *MAP3K7*, suggesting that their re-expression may attenuate TAK1-driven NF-κB and MAPK activation, consistent with earlier functional studies [90,91,92,93]. Similarly, our data showed that miR-95-5p was downregulated by LPS but increased with adalimumab, exhibiting an inverse relationship with *CXCL2* expression—strongly supporting a functional miRNA–mRNA interaction [94].

We also identified reciprocal regulation of *TNFAIP3* and miR-125b: while *TNFAIP3* increased under LPS and decreased with adalimumab, miR-125b displayed the opposite trend. This aligns with previous reports describing miR-125b as a regulator of immune signaling [95], but our findings demonstrate its involvement specifically in keratinocyte responses to adalimumab [96,97,98,99].

Conversely, we found that miR-4329 was induced by LPS and suppressed after adalimumab treatment, consistent with its predicted regulation of *XIAP* [100].

Similarly, miR-20b-3p, which targets *BIRC3*, was upregulated by LPS and reduced by adalimumab [101,102]. These results support a model where adalimumab resets the miRNA landscape by enhancing anti-inflammatory and pro-apoptotic miRNAs, while reducing those that sustain chronic inflammation [57,87,88,89].

GO enrichment analysis confirmed that the downregulated DEGs were enriched in classical inflammatory pathways, including IκB kinase/NF-κB, TNF-mediated, and Toll-like receptor signaling. No significantly enriched processes were detected among upregulated DEGs, indicating that the predominant effect of adalimumab in this model is suppression of central pro-inflammatory hubs. This selective pattern suggests that adalimumab does not act as a blanket suppressor but specifically reprograms keratinocyte signaling toward resolution of inflammation.

Systems-level STRING analysis supported this interpretation: our PPI network revealed a highly interconnected NF-κB module with IKBKB as a central hub, coordinating immune and apoptotic signals [103]. Functional enrichment analysis [104] identified key processes such as immune regulation, cellular response to LPS, and regulation of apoptosis—hallmarks of psoriatic pathology. Our data demonstrate that adalimumab disrupts this pathological circuitry modulating both inflammatory effectors and apoptosis regulators, thereby promoting a shift toward a homeostatic keratinocyte phenotype [52,83,84,85,86]. Despite these encouraging findings, several limitations must be acknowledged. Most notably, our in vitro keratinocyte model does not capture the multicellular complexity of psoriatic skin, where dynamic interactions among keratinocytes, T cells, dendritic cells, and fibroblasts critically influence disease maintenance and therapeutic response. While our results provide valuable mechanistic insight at the keratinocyte level, further validation in ex vivo skin models, three-dimensional organotypic cultures, or patient-derived organoids is warranted to confirm translational relevance.

Moreover, although we identified several miRNA–mRNA correlations supporting post-transcriptional regulation of NF-κB signaling—such as luciferase reporter assays, miRNA mimic or inhibitor experiments, and loss- or gain-of-function studies— functional validation was not performed and will be necessary to establish direct causal interactions.

Finally, the STRING database was employed as a predictive tool to explore networks among NF-κB-related differentially expressed genes. While STRING provides a useful preliminary framework for mapping potential molecular relationships, its computational predictions should be experimentally verified. Future research should therefore incorporate biochemical assays such as co-immunoprecipitation (Co-IP) or pull-down assays using tagged fusion proteins (e.g., GST-tag, His-tag) to confirm the physical binding of key NF-κB regulators, including IKBKB, TRAF2, and MAP3K7. Combining these validation approaches with proteomic or phospho-proteomic analyses would strengthen the mechanistic interpretation of adalimumab-mediated NF-κB modulation in keratinocytes. In conclusion, our findings indicate that adalimumab exerts a two-pronged effect: suppression of NF-κB–driven inflammatory circuits and reinforcement of regulatory processes that preserve keratinocyte viability. This systems-level reprogramming may explain its sustained clinical efficacy in psoriasis and supports NF-κB–associated miRNAs as potential biomarkers of therapeutic response. Future studies in ex vivo skin models and longitudinal patient cohorts will be essential to validate these mechanisms and assess their translational relevance.

## 4. Materials and Methods

This section builds upon findings from our previous studies [53,55,56].

### 4.1. Study Design

This study investigated the modulatory effects of adalimumab on NF-κB–related signaling in HaCaT human keratinocytes under inflammatory stress induced by lipopolysaccharide (LPS). A multilevel approach combining transcriptomic (mRNA microarray), post-transcriptional (miRNA microarray), and protein (ELISA) analyses was used to characterize temporal changes at 2 h, 8 h, and 24 h following LPS and adalimumab exposure. Experimental procedures are summarized in Figure 1.

### 4.2. Keratinocyte Cell Culture

HaCaT cells (AddexBio, San Diego, CA, USA) were cultured in Dulbecco’s Modified Eagle Medium (DMEM; Gibco, Thermo Fisher Scientific, Waltham, MA, USA) supplemented with 10% fetal bovine serum (FBS; Gibco), 100 U/mL penicillin, and 100 μg/mL streptomycin (Sigma-Aldrich, St. Louis, MO, USA). Cells were maintained at 37 °C in a humidified atmosphere with 5% CO_2_.

For experiments, cells were seeded at 1 × 10^6^ per well and allowed to reach 80% confluence. The inflammatory model was established using LPS from Escherichia coli (1 µg/mL; Sigma-Aldrich). Adalimumab (Humira^®^, AbbVie, Chicago, IL, USA) was added at a final concentration of 8 µg/mL, consistent with therapeutic serum levels. Experimental groups were as follows: untreated control (C), LPS-stimulated (LPS), and LPS + adalimumab–treated at 2 h (H_2_), 8 h (H_8_), and 24 h (H_24_).

Cytotoxicity was assessed in a preliminary experiment using the sulforhodamine B (SRB) assay (Sigma-Aldrich, Cat. No. 3520-42-1). HaCaT cells were treated with LPS (1, 2, or 10 μg/mL) or adalimumab (0.8, 8, or 80 μg/mL) for 24 h, and absorbance was measured at 490–530 nm. Viability was expressed as a percentage relative to untreated controls [56].

### 4.3. Total Ribonucleic Acid (RNA) Extraction

Total ribonucleic acid (RNA) was isolated from untreated HaCaT cells (control, C), LPS-stimulated cells (LPS), and LPS-primed cells subsequently treated with adalimumab for 2, 8, or 24 h (H_2_, H_8_, H_24_). Extraction was performed using TRIzol reagent (Invitrogen Life Technologies, Carlsbad, CA, USA; Cat. No. 15596026) in accordance with the manufacturer’s protocol. To improve RNA quality and remove residual contaminants, samples were further purified using the RNeasy Mini Kit (QIAGEN, Hilden, Germany; Cat. No. 74104) combined with on-column DNase I digestion (Thermo Fisher Scientific; Cat. No. 18047019) to eliminate genomic DNA. Purified RNA was eluted in RNase-free water to a final volume of 100 µL. Concentrations ranged between 80 and 150 ng/µL, and A260/A280 absorbance ratios exceeded 1.8 in all samples as assessed by NanoDrop spectrophotometry (Thermo Fisher Scientific, Waltham, MA, USA), confirming sufficient yield and purity for downstream analyses.

### 4.4. NF-κB Gene Expression Profiling via Oligonucleotide Microarrays

Global mRNA expression profiling was conducted to investigate transcriptional changes in NF-κB–related genes. HG-U133_A2 oligonucleotide microarrays (Affymetrix, Santa Clara, CA, USA) and the GeneChip™ 3′ IVT PLUS Reagent Kit (Affymetrix; Cat. No. 902416) were used according to the manufacturer’s instructions. The NF-κB pathway gene set (hsa04064) was obtained from the Kyoto Encyclopedia of Genes and Genomes (KEGG; accessed 14 April 2021), yielding 105 pathway-associated transcripts [105,106,107]. Samples were collected from untreated control cells (C), LPS-only cells (8 h), and adalimumab-treated groups after 8 h of LPS priming followed by 2, 8, or 24 h of adalimumab exposure (H_2_, H_8_, H_24_). Each experimental group was analyzed in three independent biological replicates. Differential expression analysis was performed between LPS and C to establish the inflammatory baseline, and between H_2_/H_8_/H_24_ and LPS to determine the effects of adalimumab. Genes were defined as differentially expressed (DEGs) if they met the threshold of |fold change| ≥ 2.0 with an adjusted *p*-value < 0.05.

### 4.5. Identification of miRNAs Potentially Regulating NF-κB Gene Expression Using miRNA Microarrays

To identify microRNAs potentially regulating NF-κB–associated transcripts, miRNA expression was profiled using GeneChip™ miRNA 2.0 arrays (Affymetrix). Experimental conditions mirrored the mRNA analysis (C, LPS, H_2_, H_8_, H_24_), with each group performed in biological triplicates. Putative regulatory interactions were evaluated using TargetScan and miRanda databases (accessed 14 April 2021) [108] Only miRNA–mRNA pairs with prediction scores ≥ 80 were retained as high-confidence interactions [108,109].

### 4.6. RT-qPCR

RT-qPCR was performed to validate expression changes in selected transcripts identified in the microarray screen. Seven genes were analyzed: *BCL2 Like 1 (BCL2L1)*, *C-X-C Motif Chemokine Ligand 2 (CXCL2)*, *Mitogen-Activated Protein Kinase Kinase Kinase 7 (MAP3K7)*, *Baculoviral IAP Repeat Containing 2 (BIRC2)*, *Baculoviral IAP Repeat Containing 3 (BIRC3)*, *TNF Alpha Induced Protein 3 (TNFAIP3)* and *X-Linked Inhibitor of Apoptosis (XIAP)*. *ACTB* (β-actin) was used as the reference gene. Reactions were carried out with the SensiFAST SYBR^®^ kit (Bioline, London, UK) and gene-specific primers (Table 4). Each sample was assayed in technical triplicates.

The qPCR protocol consisted of reverse transcription at 45 °C for 10 min, followed by 40 amplification cycles. Specificity of amplification products was confirmed by melting curve analysis and agarose gel electrophoresis. Relative expression was calculated using the 2^−ΔΔCt^ method [110]. All time points and experimental groups (C, LPS, H_2_, H_8_, H_24_) were included in the analysis, each in biological triplicates.

### 4.7. Protein Quantification by ELISA

To examine post-transcriptional regulation, protein levels of BCL2L1, CXCL2, MAP3K7, BIRC2, BIRC3, TNFAIP3, and XIAP were quantified by ELISA. HaCaT cells from all groups (C, LPS, H_2_, H_8_, H_24_) were harvested, washed twice with ice-cold PBS, and lysed in RIPA buffer. Lysates were centrifuged at 12,000× *g* for 10 min at 4 °C, and supernatants were collected for analysis.

Commercial ELISA kits (MyBioSource, San Diego, CA, USA) specific for each target protein were used according to the manufacturer’s protocols: BCL2L1 (MBS9392826), CXCL2 (MBS2880010), MAP3K7 (MBS7612749), TNFAIP3 (MBS2881407), XIAP (MBS161168), BIRC2 (MBS9426546), and BIRC3 (MBS7235955). Standard curves were generated using recombinant protein standards, and absorbance was measured with a microplate reader. Protein concentrations were calculated from standard curves and normalized to total protein content. All assays were conducted in biological triplicates.

### 4.8. Statistical Analysis

Data analysis was performed using the Transcriptome Analysis Console (Thermo Fisher Scientific, USA) and StatPlus Pro v7.3 (AnalystSoft Inc., Brandon, FL, USA). The distribution of all variables was assessed with the Shapiro–Wilk test, and for data meeting the assumptions of normality, one-way ANOVA was applied. In the case of microarray analysis, differentially expressed genes (DEGs) were defined as those with an absolute fold change of at least 2.0 and an adjusted *p*-value below 0.05. Comparisons were made between LPS-stimulated cells and untreated controls (C) to establish the inflammatory baseline, as well as between adalimumab-treated groups (H_2_, H_8_, H_24_) and the LPS group to determine drug-specific effects. Tukey’s HSD post hoc test was used for multiple comparisons in the microarray dataset. For RT-qPCR and ELISA analyses, expression differences among groups were evaluated using one-way ANOVA followed by Scheffe’s post hoc test, which was selected for its robustness in cases of unequal group sizes. Results are presented as mean values with standard error of the mean (SEM), and statistical significance was defined as *p* < 0.05.

#### Protein–Protein Interaction (PPI) Networks and Gene Ontology (GO) Enrichment Analyses

Functional interpretation of DEGs was performed using STRING version 12.0 (https://string-db.org, accessed on 1 June 2025) [111]. A high-confidence interaction threshold (minimum required score 0.7) was applied. Both experimentally validated interactions and predicted associations (gene neighborhood, co-occurrence, and text mining) were included. Gene Ontology Biological Process (GO:BP) enrichment was performed on the combined set of DEGs from Table 2 (union across time points and conditions, regardless of direction of regulation). STRING output included network visualization, significantly enriched GO:BP terms, and associated false discovery rate (FDR)-corrected *p*-values.

## 5. Conclusions

This study elucidates the multifaceted molecular impact of adalimumab in LPS-stimulated keratinocytes, demonstrating its ability to restore inflammatory homeostasis through coordinated suppression of NF-κB and MAPK signaling and modulation of key regulatory microRNAs, including miR-125b, miR-95-5p, and miR-30a. The reciprocal expression patterns between these miRNAs and their validated targets—*TNFAIP3*, *CXCL2*, and *MAP3K7*—highlight a coherent post-transcriptional regulatory network responsive to TNF-α inhibition. In parallel, the regulation of apoptosis-related genes such as *BIRC3* and *XIAP* underscores adalimumab’s dual role in modulating keratinocyte survival and dampening immune activation. Collectively, these findings advance our mechanistic understanding of biologic therapy in psoriasis and identify a panel of miRNAs as potential biomarkers for treatment monitoring and patient stratification. Future translational studies and clinical trials integrating miRNA profiling with multi-omics approaches are warranted to validate these targets and to pave the way toward more precise, individualized therapeutic strategies in inflammatory skin diseases.

## Figures and Tables

**Table 1:** Selected protein-coding genes differentially expressed under LPS and/or adalimumab treatment in HaCaT keratinocytes.

ID	mRNA	LPS vs. C	H_2 vs. LPS	H_8 vs. LPS	H_24 vs. LPS
1563357_at		(+)6.17	(−)3.41	(−)3.81	(−)3.04
206665_s_at	*BCL2L1*	(+)3.98	(−)2.11	(−)2.98	(−)2.01
212312_at		(+)3.28	(−)2.12	(−)2.71	(−)2.09
215037_s_at		(+)3.21	(−)2.18	(−)2.91	(−)2.07
231228_at		(+)3.18	(−)2.13	(−)3.01	(−)2.11
206853_s_at	*MAP3K7*	(+)3.98	(−)2.91	(−)3.59	(−)3.11
206854_s_at		(+)4.19	(−)2.99	(−)3.61	(−)3.01
211536_x_at		(+)3.99	(−)2.91	(−)3.44	(−)3.17
211537_x_at		(+)3.76	(−)2.92	(−)3.49	(−)3.11
202076_at	*BIRC2*	(+)2.12	(−)4.54	(−)4.19	(−)3.01
210538_s_at	*BIRC3*	(+)2.12	(−)4.14	(−)4.76	(−)3.22
230499_at		(+)2.11	(−)3.17	(−)4.91	(−)3.19
202643_s_at	*TNFAIP3*	(+)3.09	(−)3.81	(−)3.31	(−)2.19
202644_s_at		(+)3.01	(−)3.89	(−)3.48	(−)2.18
206536_s_at	*XIAP*	(+)2.81	(−)3.44	(−)2.91	(−)2.11
206537_at		(+)2.76	(−)3.43	(−)2.99	(−)2.18
225858_s_at		(+)2.91	(−)3.71	(−)2.98	(−)2.17
225859_at		(+)2.81	(−)3.32	(−)2.78	(−)2.16
228363_at		(+)2.54	(−)3.54	(−)3.02	(−)2.11
235222_x_at		(+)2.79	(−)3.45	(−)2.89	(−)2.24
243026_x_at		(+)2.80	(−)3.19	(−)2.99	(−)2.08
209774_x_at	*CXCL2*	(+)2.19	(−)2.45	(−)2.19	(−)2.01
230101_at		(+)2.18	(−)2.31	(−)2.11	(−)2.03
1569203_at		(+)2.11	(−)2.49	(−)2.18	(−)2.03

Data represent fold changes (mean ± SEM, *n* = 3) from microarray analysis. Genes were considered differentially expressed at |fold change| ≥ 2.0 and adjusted *p* < 0.05. Comparisons: LPS vs. C (baseline inflammatory response); H_2_, H_8_, H_24_ vs. LPS (adalimumab-specific effects). Statistical analysis: one-way ANOVA with Tukey’s HSD post hoc test. Abbreviations: (+), upregulation relative to the comparator group; (−), downregulation relative to the comparator group. In comparisons of LPS vs. C, symbols indicate regulation relative to untreated controls. In comparisons of H_2_, H_8_, and H_24_ vs. LPS; C, untreated control; LPS, lipopolysaccharide-stimulated cells; H_2_, H_8_, H_24_, adalimumab treatment for 2, 8, and 24 h, respectively.

**Table 2:** Differential expression of microRNAs predicted to regulate selected NF-κB–related mRNAs in HaCaT keratinocytes (*p* < 0.05; FC > 2 or < −2).

mRNA	miRNA	Target Score	Fold Change			
			LPS vs. C	H_2 vs. LPS	H_8 vs. LPS	H_24 vs. LPS
*MAP3K7*	miR-1297	90	(−)2.13 ± 0.18 *	(+)2.18 ± 0.19 *	(+)2.11 ± 0.19 *	(+)2.87 ± 0.98 *
	miR-30a	83	(−)2.17 ± 0.12 *	(+)2.17 ± 0.81 *	(+)2.45 ± 0.18 *	(+)2.54 ± 0.12 *
*CXCL2*	miR-95-5p	96	(−)3.12 ± 0.45 *	(+)2.87 ± 0.81 *	(+)3.12 ± 0.91 *	(+)2.10 ± 0.53 *
*TNFAIP3*	miR-125b	84	(−)2.18 ± 0.13 *	(+)2.56 ± 0.43 *	(+)2.11 ± 0.71 *	(+)2.03 ± 0.71 *
*XIAP*	miR-4329	94	(+)2.98 ± 0.76 *	(−)2.11 ± 0.71 *	(−)2.76 ± 0.76 *	(−)2.98 ± 0.13 *
*BIRC3*	mir-20b-3p	96	(+)3.18 ± 0.55 *	(−)2.18 ± 0.12 *	(−)2.11 ± 0.43 *	(−)2.16 ± 0.41 *

Data are presented as fold change (mean ± SEM, *n* = 3). Criteria for differential expression: |fold change| ≥ 2.0, *p* < 0.05. Comparisons: LPS vs. C (inflammatory induction); H_2_, H_8_, H_24_ vs. LPS (adalimumab effects). Statistical analysis: one-way ANOVA with Scheffé’s post hoc test. Abbreviations: (+), upregulation relative to the comparator group; (−), downregulation relative to the comparator group. In comparisons of LPS vs. C, symbols indicate regulation relative to untreated controls. In comparisons of H_2_, H_8_, and H_24_ vs. LPS, symbols indicate regulation relative to LPS-stimulated cells. *, *p* < 0.05. Gene abbreviations: *CXCL2*, C-X-C Motif Chemokine Ligand 2; *MAP3K7*, Mitogen-Activated Protein Kinase Kinase Kinase 7; *BIRC3,* Baculoviral IAP Repeat Containing 3; *TNFAIP3*, Tumor Necrosis Factor Alpha-Induced Protein 3; *XIAP*, X-Linked Inhibitor of Apoptosis Protein.

**Table 3:** Concentrations of NF-κB– and MAPK-related proteins in HaCaT culture supernatants following LPS stimulation and subsequent adalimumab treatment for 2, 8, and 24 h, measured by ELISA.

Protein	C	LPS	H_2	H_8	H_24
BCL2L1 [ng/mL]	13.19 ± 0.23	87.12 ± 19 *	18.13 ± 0.16 *	17.87 ± 0.65 *	17.19 ± 0.18 *
CXCL2 [ng/mL]	1.56 ± 0.91	7.13 ± 1.09 *	4.23 ± 0.65 *	3.34 ± 0.51 *	2.19 ± 0.81 *
MAP3K7 [ng/mL]	2.91 ± 0.34	6.77 ± 1.12 *	4.56 ± 0.91 *	2.87 ± 0.51 *	2.11 ± 0.13 *
BIRC2 [pg/mL]	846.12 ± 5.71	2454.12 ± 105.91 *	176.12 ± 3.67 *	1056 ± 9.12 *	1023 ± 9.81 *
BIRC3 [pg/mL]	961.10 ± 32.98	3871.91 ± 176.65 *	2098 ± 18.24 *	2012 ± 14.81 *	2019 ± 21.91 *
TNFAIP3 [ng/mL]	6.18 ± 0.98	19.87 ± 1.76 *	12.12 ± 2.17 *	11.62 ± 0.98 *	10.01 ± 0.12 *
XIAP [ng/mL]	3.48 ± 0.91	7.65 ± 0.76 *	5.57 ± 1.11 *	6.12 ± 0.91 *	6.80 ± 0.19 *

C, control culture; Data are presented as mean ± SEM from three independent biological replicates (*n* = 3). Comparisons: LPS vs. C (inflammatory induction); H_2_, H_8_, H_24_ vs. LPS (adalimumab effects). Statistical analysis: one-way ANOVA with Scheffé’s post hoc test. In comparisons of LPS vs. C, symbols indicate regulation relative to untreated controls. In comparisons of H_2_, H_8_, and H_24_ vs. LPS, symbols indicate regulation relative to LPS-stimulated cells. *, *p* < 0.05; C, untreated control; LPS, lipopolysaccharide-stimulated cells; H_2_, H_8_, H_24_, adalimumab treatment for 2, 8, and 24 h, respectively. Protein abbreviations: BCL2L1, BCL2 Like 1; CXCL2, C-X-C Motif Chemokine Ligand 2; MAP3K7, Mitogen-Activated Protein Kinase Kinase Kinase 7; BIRC2, Baculoviral IAP Repeat Containing 2; BIRC3, Baculoviral IAP Repeat Containing 3; TNFAIP3, Tumor Necrosis Factor Alpha-Induced Protein 3; XIAP, X-Linked Inhibitor of Apoptosis Protein.

**Table 4:** RT-qPCR primers.

mRNA	RT-qPCR Primers (5′-3′)
*BCL2L1*	Forward: GCCACTTACCTGAATGACCACC Reverse: AACCAGCGGTTGAAGCGTTCCT
*CXCL2*	Forward: GGCAGAAAGCTTGTCTCAACCC Reverse: CTCCTTCAGGAACAGCCACCAA
*MAP3K7*	Forward: CAGAGCAACTCTGCCACCAGTA Reverse: CATTTGTGGCAGGAACTTGCTCC
*BIRC2*	Forward: CTGTGGTGGGAAGCTCAGTA Reverse: TCATTCGAGCTGCATGTGTC
*BIRC3*	Forward: GGCTGTTACCGCTGAGAATG Reverse: C GGTGGCAGGAGAAACATCA
*TNFAIP3*	Forward: CTCAACTGGTGTCGAGAAGTCC Reverse: TTCCTTGAGCGTGCTGAACAGC
*XIAP*	Forward: TGGCAGATTATGAAGCACGGATC Reverse: AGTTAGCCCTCCTCCACAGTGA
*ACTB*	Forward: TCACCCACACTGTGCCCATCTACGA Reverse: CAGCGGAACCGCTCATTGCCAATGG

Gene Abbreviations: *BCL2L1*, BCL2 like 1; *CXCL2*, C-X-C motif chemokine ligand 2; *MAP3K7*, mitogen-activated protein kinase kinase kinase 7; *BIRC2*, baculoviral IAP repeat-containing protein 2; *BIRC3*, baculoviral IAP repeat-containing protein 3; *TNFAIP3*, tumor necrosis factor alpha-induced protein 3; *XIAP*, X-linked inhibitor of apoptosis protein; *ACTB*, β-actin.

## Data Availability

The data presented in this study are available in this article.

## Supplementary Materials

The following supporting information can be downloaded at: https://www.mdpi.com/article/10.3390/ijms262010035/s1.

## Abbreviations

The following abbreviations are used in this manuscript:

**Abbreviation**	**Full Term**
NF-κB	Nuclear factor kappa B
TNF-α	Tumor necrosis factor alpha
miRNA	MicroRNA
LPS	Lipopolysaccharide
DEG	Differentially expressed gene
RT-qPCR	Reverse transcription quantitative polymerase chain reaction
ELISA	Enzyme-linked immunosorbent assay
PPI	Protein–protein interaction
GO	Gene Ontology
HaCaT	Human immortalized keratinocyte
GEO	Gene Expression Omnibus
KEGG	Kyoto Encyclopedia of Genes and Genomes
CXCL2	C-X-C motif chemokine ligand 2
IRAK1	Interleukin-1 receptor–associated kinase 1
MAP3K7	Mitogen-activated protein kinase kinase kinase 7 (TAK1)
BIRC2/3	Baculoviral inhibitor of apoptosis repeat-containing protein 2/3
XIAP	X-linked inhibitor of apoptosis protein
TNFAIP3	Tumor necrosis factor alpha-induced protein 3 (A20)
IKBKB	Inhibitor of nuclear factor kappa-B kinase subunit beta (IKKβ)
SYK	Spleen tyrosine kinase
TAB1	TGF-beta-activated kinase 1/MAP3K7 binding protein 1
TAB2	TGF-beta-activated kinase 1/MAP3K7 binding protein 2
PASI	Psoriasis Area and Severity Index
JAK/STAT	Janus kinase/signal transducer and activator of transcription
MAPK	Mitogen-activated protein kinase
STAT3	Signal transducer and activator of transcription 3
AP-1	Activator protein 1
FDR	False discovery rate
DMEM	Dulbecco’s Modified Eagle Medium
FBS	Fetal bovine serum
PBS	Phosphate-buffered saline
RIPA	Radioimmunoprecipitation assay
STRING	Search Tool for the Retrieval of Interacting Genes/Proteins

## Appendix A

**Table A1:** GO Biological Process enrichment for proteins encoded by genes listed in Table 2 (union set; STRING analysis, FDR < 0.05).

ID	Term Description	Observed Gene Count	Background Gene Count	Strength	Signal	False Discovery Rate	Proteins in Network
GO:0038095	Fc-epsilon receptor signaling pathway	7	23	2.48	4.92	2.65 × 10^−12^	PLCG1, MAP3K7, SYK, IKBKB, TRAF6, PLCG2, BTK
GO:0050851	Antigen receptor-mediated signaling pathway	8	141	1.75	3.08	6.22 × 10^−10^	MAPK1, PLCG1, MAP3K7, SYK, IKBKB, TRAF6, PLCG2, BTK
GO:0038061	NIK/NF-kappaB signaling	6	36	2.22	3.67	1.16 × 10^−9^	TRAF2, BIRC3, MAP3K7, IRAK1, TRAF6, BIRC2
GO:0007249	I-kappaB kinase/NF-kappaB signaling	7	68	2.01	3.55	5.05 × 10^−10^	TRAF2, BIRC3, TAB2, MAP3K7, IRAK1,TNF, IKBKB, TRAF6, BIRC2, TNFAIP3, PLCG2
GO:0002223	Stimulatory C-type lectin receptor signaling pathway	5	22	2.35	3.36	1.42 × 10^−8^	MAP3K7, SYK, IKBKB, TRAF6, PLCG2
GO:0043122	Regulation of I-kappaB kinase/NF-kappaB signaling	11	255	1.63	3.29	2.45 × 10^−12^	TRAF2, BIRC3, TAB2, MAP3K7, IRAK1, TNF, IKBKB, TRAF6, BIRC2, TNFAIP3, PLCG2
GO:0043123	Positive regulation of I-kappaBkinase/NF-kappaB signaling	9	191	1.67	3.05	1.51 × 10^−10^	TRAF2, BIRC3, TAB2, MAP3K7, IRAK1, TNF, IKBKB, TRAF6, BIRC2
GO:0033209	Tumor necrosis factor-mediatedsignaling pathway	6	56	2.02	3.16	9.38 × 10^−9^	TRAF2, BIRC3, TNF, IKBKB, TRAF6, BIRC2
GO:0002224	Toll-like receptor signaling pathway	6	61	1.99	3.06	1.38 × 10^−8^	MAP3K7, IRAK1, TNF, TRAF6, PLCG2, BTK
GO:0002221	Pattern recognition receptor signalingpathway	7	101	1.83	3.04	3.66 × 10^−9^	MAP3K7, IRAK1, TNF, TRAF6, TNFAIP3, PLCG2, BTK

Protein abbreviations: IKBKB, Inhibitor of Nuclear Factor Kappa B Kinase Subunit Beta; TRAF2, TNF Receptor Associated Factor 2; IRAK1, Interleukin-1 Receptor-Associated Kinase 1; SYK, Spleen Tyrosine Kinase; PLAU, Plasminogen Activator, Urokinase; TAB2, TGF-Beta Activated Kinase 1 (MAP3K7) Binding Protein 2; MAPK14, Mitogen-Activated Protein Kinase 14; PLCG1, Phospholipase C Gamma 1; PLCG2, Phospholipase C Gamma 2; BTK, Bruton’s Tyrosine Kinase; TRAF6, TNF Receptor Associated Factor 6; TAB1, TGF-Beta Activated Kinase 1 (MAP3K7) Binding Protein 1; TNF, Tumor Necrosis Factor; BCL2L1, BCL2 Like 1; CXCL2, C-X-C Motif Chemokine Ligand 2; MAP3K7, Mitogen-Activated Protein Kinase Kinase Kinase 7; BIRC2, Baculoviral IAP Repeat Containing 2; BIRC3, Baculoviral IAP Repeat Containing 3; TNFAIP3, Tumor Necrosis Factor Alpha-Induced Protein 3; XIAP, X-Linked Inhibitor of Apoptosis Protein.
