# Supplementary material for: Modulation of Nuclear Factor Kappa B Signaling and microRNA Profiles by Adalimumab in LPS-Stimulated Keratinocytes
Source: Int J Mol Sci. 2025 Oct 15;26(20):10035. doi: 10.3390/ijms262010035 (PMC12563313; doi:10.3390/ijms262010035)
Supplement: Supplementary file 1 [file ijms-26-10035-s001.zip › ijms-3914264-supplementary.pdf]

Table S1. Transcriptional activity of NF-κB–related genes in HaCaT keratinocytes following LPS stimulation and subsequent adalimumab treatment at 2, 8, and 24 hours compared to control cultures ($p < 0.05$).

ID	mRNA	LPS vs. C	H_2 vs. LPS	H_8 vs. LPS	H_24 vs. LPS
209341_s_at	*IKBKB*	(+)3.14	(−)2.87	(−)2.18	(−)2.09
209342_s_at		(+)3.15	(−)2.38	(−)2.12	(−)2.12
211027_s_at		(+)3.21	(−)2.81	(−)2.19	(−)2.15
204413_at	*TRAF2*	(+)2.19	(−)2.34	(−)2.33	(−)2.18
201587_s_at	*IRAK1*	(+)3.41	(−)2.45	(−)2.19	(−)2.11
1555784_s_at		(+)3.13	(−)2.19	(−)2.24	(−)2.08
207540_s_at	*SYK*	(+)4.31	(−)2.17	(−)2.23	(−)2.12
209269_s_at		(+)4.14	(−)2.18	(−)2.32	(−)2.15
226068_at		(+)4.51	(−)2.11	(−)2.31	(−)2.11
244023_at		(+)4.09	(−)2.13	(−)2.19	(−)2.09
205479_s_at	*PLAU*	(+)2.10	(−)3.19	(−)2.12	(−)2.76
211668_s_at		(+)2.19	(−)3.21	(−)2.18	(−)2.71
210284_s_at	*TAB2*	(+)3.13	(−)3.09	(−)3.19	(−)2.54
212184_s_at		(+)3.19	(−)3.12	(−)3.19	(−)2.49
1561300_at		(+)3.45	(−)3.10	(−)3.31	(−)2.61
MAPK1	*MAPK1*	(+)5.98	(−)2.11	(−)3.98	(−)4.01
MAPK1		(+)5.81	(−)2.18	(−)3.71	(−)4.08
MAPK1		(+)5.91	(−)2.19	(−)3.91	(−)4.19
MAPK1		(+)5.67	(−)2.12	(−)4.02	(−)4.04
MAPK1		(+)5.71	(−)2.17	(−)3.99	(−)4.07
MAPK1		(+)5.41	(−)2.23	(−)3.84	(−)4.04
MAPK1		(+)5.10	(−)2.21	(−)3.91	(−)4.03
202789_at	*PLCG1*	(+)4.12	(−)2.98	(−)3.76	(−)3.32
216551_x_at		(+)4.19	(−)2.34	(−)3.72	(−)3.29
240805_at		(+)4.19	(−)2.45	(−)3.43	(−)3.19
204613_at	*PLCG2*	(+)3.14	(−)2.71	(−)2.91	(−)2.11
230917_at		(+)3.87	(−)2.18	(−)2.71	(−)2.32
1557993_at		(+)3.43	(−)2.28	(−)2.82	(−)2.19
205504_at	*BTK*	(+)2.81	(−)2.11	(−)2.32	(−)2.12
205558_at	*TRAF6*	(+)2.53	(−)3.19	(−)3.91	(−)2.11
227264_at		(+)2.54	(−)3.32	(−)3.43	(−)2.13
203901_at	*TAB1*	(+)2.81	(−)2.01	(−)2.11	(−)2.08
207113_s_at	*TNF*	(+)6.19	(−)3.74	(−)3.91	(−)3.01
1563357_at		(+)6.17	(−)3.41	(−)3.81	(−)3.04
206665_s_at	*BCL2L1*	(+)3.98	(−)2.11	(−)2.98	(−)2.01
212312_at		(+)3.28	(−)2.12	(−)2.71	(−)2.09
215037_s_at		(+)3.21	(−)2.18	(−)2.91	(−)2.07
231228_at		(+)3.18	(−)2.13	(−)3.01	(−)2.11
206853_s_at	*MAP3K7*	(+)3.98	(−)2.91	(−)3.59	(−)3.11
206854_s_at		(+)4.19	(−)2.99	(−)3.61	(−)3.01
211536_x_at		(+)3.99	(−)2.91	(−)3.44	(−)3.17
211537_x_at		(+)3.76	(−)2.92	(−)3.49	(−)3.11

202076_at	*BIRC2*	(+)2.12	(−)4.54	(−)4.19	(−)3.01
210538_s_at	*BIRC3*	(+)2.12	(−)4.14	(−)4.76	(−)3.22
230499_at		(+)2.11	(−)3.17	(−)4.91	(−)3.19
202643_s_at	*TNFAIP3*	(+)3.09	(−)3.81	(−)3.31	(−)2.19
202644_s_at		(+)3.01	(−)3.89	(−)3.48	(−)2.18
206536_s_at	*XIAP*	(+)2.81	(−)3.44	(−)2.91	(−)2.11
206537_at		(+)2.76	(−)3.43	(−)2.99	(−)2.18
225858_s_at		(+)2.91	(−)3.71	(−)2.98	(−)2.17
225859_at		(+)2.81	(−)3.32	(−)2.78	(−)2.16
228363_at		(+)2.54	(−)3.54	(−)3.02	(−)2.11
235222_x_at		(+)2.79	(−)3.45	(−)2.89	(−)2.24
243026_x_at		(+)2.80	(−)3.19	(−)2.99	(−)2.08
209774_x_at	*CXCL2*	(+)2.19	(−)2.45	(−)2.19	(−)2.01
230101_at		(+)2.18	(−)2.31	(−)2.11	(−)2.03
1569203_at		(+)2.11	(−)2.49	(−)2.18	(−)2.03

Data represent fold changes (mean ± SEM, n = 3) from microarray analysis. Genes were considered differentially expressed at |fold change| ≥ 2.0 and adjusted $p < 0.05$. Comparisons: LPS vs. C (baseline inflammatory response); H_2, H_8, H_{24} vs. LPS (adalimumab-specific effects). Statistical analysis: one-way ANOVA with Tukey’s HSD post hoc test. Abbreviations: (+), upregulation relative to the comparator group; (−), downregulation relative to the comparator group. In comparisons of LPS vs. C, symbols indicate regulation relative to untreated controls. In comparisons of H_2, H_8, and H_{24} vs. LPS; C, untreated control; LPS, lipopolysaccharide-stimulated cells; H_2, H_8, H_{24}, adalimumab treatment for 2, 8, and 24 h, respectively.

Gene abbreviations: *IKBKB*, Inhibitor of Nuclear Factor Kappa B Kinase Subunit Beta; *TRAF2*, TNF Receptor Associated Factor 2; *IRAK1*, Interleukin-1 Receptor-Associated Kinase 1; *SYK*, Spleen Tyrosine Kinase; *PLAU*, Plasminogen Activator, Urokinase; *TAB2*, TGF-Beta Activated Kinase 1 (MAP3K7) Binding Protein 2; *MAPK14*, Mitogen-Activated Protein Kinase 14; *PLCG1*, Phospholipase C Gamma 1; *PLCG2*, Phospholipase C Gamma 2; *BTK*, Bruton's Tyrosine Kinase; *TRAF6*, TNF Receptor Associated Factor 6; *TAB1*, TGF-Beta Activated Kinase 1 (MAP3K7) Binding Protein 1; *TNF*, Tumor Necrosis Factor; *BCL2L1*, B-cell lymphoma-extra-large; *CXCL2*, C-X-C Motif Chemokine Ligand 2; *MAP3K7*, Mitogen-Activated Protein Kinase Kinase Kinase 7; *BIRC2*, Baculoviral IAP Repeat Containing 2; *BIRC3*, Baculoviral IAP Repeat Containing 3; *TNFAIP3*, Tumor Necrosis Factor Alpha-Induced Protein 3; *XIAP*, X-Linked Inhibitor of Apoptosis Protein.
